# Emodin-Loaded Thermoresponsive Hydrogel as a Potential Drug Delivery System for Periodontal Disease in a Rat Model of Ligature-Induced Periodontitis

**DOI:** 10.3390/polym17152108

**Published:** 2025-07-31

**Authors:** Gyu-Yeon Shim, Seong-Hee Moon, Seong-Jin Shin, Hyun-Jin Kim, Seunghan Oh, Ji-Myung Bae

**Affiliations:** 1 Department of Dental Biomaterials, College of Dentistry, Wonkwang University, Iksan 54538, Jeonbuk, Republic of Korea; koala9710@naver.com (G.-Y.S.); ko2742@naver.com (S.-J.S.); shoh@wku.ac.kr (S.O.); 2 Institute of Biomaterials and Implant, College of Dentistry, Wonkwang University, Iksan 54538, Jeonbuk, Republic of Korea; shmoon@jif.re.kr (S.-H.M.); khjin1005@wku.ac.kr (H.-J.K.); 3 Jeonbuk Institute for Food Bio-Industry, Jeonju 54810, Jeonbuk, Republic of Korea; 4 Institute of Tissue Regeneration Engineering (ITREN), Dankook University, Cheonan 31116, Chungnam, Republic of Korea; 5 Department of Oral Anatomy, College of Dentistry, Wonkwang University, Iksan 54538, Jeonbuk, Republic of Korea; 6 Musculoskeletal and Immune Disease Research Institute, Wonkwang University, Iksan 54538, Jeonbuk, Republic of Korea

**Keywords:** emodin, thermoresponsive hydrogel, local drug delivery, periodontitis, antibacterial, anti-inflammatory

## Abstract

Periodontitis, a chronic inflammatory disease, causes alveolar bone loss. Current treatments show limitations in achieving dual antimicrobial and anti-inflammatory effects. We evaluated an emodin-loaded thermoresponsive hydrogel as a local drug delivery system for periodontitis treatment. Emodin itself demonstrated antibacterial activity against *Porphyromonas gingivalis*, with minimal inhibitory and minimal bactericidal concentrations of 50 μM. It also suppressed mRNA expression of proinflammatory cytokines [tumor necrosis factor alpha, interleukin (IL)-1β, and IL-6] in lipopolysaccharide-stimulated RAW 264.7 cells. The hydrogel, formulated with poloxamers and carboxymethylcellulose, remained in a liquid state at room temperature and formed a gel at 34 °C, providing sustained drug release for 96 h and demonstrating biocompatibility with human periodontal ligament stem cells while exhibiting antibacterial activity against *P. gingivalis*. In a rat model of periodontitis, the hydrogel significantly reduced alveolar bone loss and inflammatory responses, as confirmed by micro-computed tomography and reverse transcription quantitative polymerase chain reaction of gingival tissue. The dual antimicrobial and anti-inflammatory properties of emodin, combined with its thermoresponsive delivery system, provide advantages over conventional treatments by maintaining therapeutic concentrations in the periodontal pocket while minimizing systemic exposure. This shows the potential of emodin-loaded thermoresponsive hydrogels as effective local delivery systems for periodontitis treatment.

## 1. Introduction

Periodontitis is a chronic inflammatory disease that affects the periodontium and supporting tissues around the teeth. Characterized by the progressive destruction of alveolar bone and the periodontal ligament, periodontitis is primarily initiated by a bacterial biofilm (dental plaque) that elicits an exaggerated immune response [1]. Without proper treatment, periodontitis can lead to tooth loss and has been associated with systemic health issues, including cardiovascular disease and diabetes [2,3]. Because severe periodontitis significantly decreases the quality of life, effective treatment is an essential topic in dental research and clinical practice [4].

Conventional treatment methods for periodontitis primarily include mechanical debridement such as scaling and root planning, with surgical intervention in severe cases. Although these treatments can effectively reduce bacterial load and inflammation, their efficacy is often limited by patient compliance, anatomical challenges, and residual calculus [5]. Moreover, these methods do not fully address the need for regenerative healing of damaged tissues, leading researchers to explore alternative and adjunctive treatments [6].

Natural products for periodontitis management have gained significant interest because of their inherent bioactive properties and low incidence of adverse effects. Many plant-derived compounds exhibit anti-inflammatory, antioxidant, and antimicrobial activities, making them potential candidates for supporting periodontal health [7]. Incorporation of natural compounds into periodontal therapies could address some of the limitations of conventional treatments by providing bioactive, targeted, and sustained therapeutic effects while minimizing systemic side effects [8].

Emodin (1,3,8-trihydroxy-6-methylanthraquinone) is one such bioactive compound extracted from various traditional medicinal plants such as *Rheum palmatum* and *Polygonum cuspidatum* [9]. Known for its anti-inflammatory, antioxidant, and antimicrobial properties, emodin has shown the potential to reduce periodontal inflammation and bacterial activity [10,11,12,13]. This natural anthraquinone compound can modulate host immune responses and offer a novel approach for periodontitis management when integrated into periodontal therapies.

Local drug delivery systems have been developed for the site-specific treatment of periodontitis, allowing higher local drug concentrations with reduced systemic exposure [14,15]. These delivery systems, such as gels, fibers, and microspheres, have shown the potential to sustain therapeutic effects at affected sites [16,17]. Despite these advantages, achievement of adequate drug retention, controlled release, and biocompatibility within the periodontal pocket environment remains challenging [18].

Thermoresponsive hydrogels represent an innovative advancement in local drug delivery systems for periodontitis treatment [19,20]. These hydrogels remain in a liquid state at lower temperatures, enabling easy injection into the affected site. Then, they solidify at oral temperatures, ensuring sustained, localized drug release [21]. The unique properties of thermoresponsive hydrogels not only enhance patient comfort during application but also improve the overall efficacy of treatment by maintaining therapeutic drug levels over an extended period, potentially leading to better clinical outcomes in cases of periodontitis [22].

The *Porphyromonas gingivalis* strain ATCC 53978 (W50) is known for its notably higher virulence compared with that exhibited by other strains, resulting in accelerated periodontal tissue destruction and advanced lesions [23,24]. The rat model of ligature-induced periodontitis, which was adopted in this study, is widely used owing to its reproducibility, clinical relevance, and ease of use [25]. This model closely mimics the pathological processes in human periodontitis, including inflammation, junctional epithelium migration, and alveolar bone resorption [26]. Furthermore, the rat model provides practical benefits such as low variability, rapid disease progression, and cost-effectiveness, making it suitable for evaluation of novel periodontal therapies [25,27].

To the best of our knowledge, no previous study has investigated the use of emodin-loaded thermoresponsive hydrogels for the prevention of periodontitis. Therefore, the aim of this study was to evaluate the antibacterial and anti-inflammatory effects of emodin and the physical and biological properties of emodin-loaded thermoresponsive hydrogels as potential local drug delivery systems for the treatment of periodontitis.

## 2. Materials and Methods

### 2.1. Cell Viability Assay for Emodin

The cytotoxicity of emodin was assessed using both human periodontal ligament stem cells (hPDLSCs; Celprogen Inc., Torrance, CA, USA) and RAW 264.7 cells (ATCC, Manassas, VA, USA). Emodin (Sigma-Aldrich, St. Louis, MO, USA) powder was dissolved in dimethyl sulfoxide (VWR International, Radnor, PA, USA) at a concentration of 100 mM. The effect of emodin on cell viability was evaluated using cell counting kit-8 (CCK-8). hPDLSCs were seeded in a 96-well plate at a density of 10,000 cells/well. After 24 h of incubation, the emodin stock solution was diluted serially with Alpha Modified Eagle’s Minimum Essential Medium (α-MEM; Gibco, Waltham, MA, USA) and added to each well at concentrations ranging from 6.25 to 100 μM in a total volume of 100 μL. After 24 h of treatment, 10 μL of CCK-8 solution (Dojindo, Kumamoto, Japan) was added to each well, and absorbance at 450 nm was measured using a microplate reader.

RAW 264.7 cells were seeded in a 96-well plate at a density of 20,000 cells/well. Emodin was diluted in Dulbecco’s Modified Eagle Medium (DMEM; Gibco, Waltham, MA, USA) at concentrations ranging from 0.1 to 100 μM and subsequently applied to the cells. Subsequent steps were performed as described above. In accordance with ISO 10993-5, cell viability of 70% or higher relative to that in the negative control indicated the lack of cytotoxicity [28].

### 2.2. Anti-Inflammatory Activity of Emodin

To evaluate the anti-inflammatory effects of emodin, RAW 264.7 cells were seeded in 24-well plates at a density of 2 × 10^5^ cells/well. After 24 h of incubation, the cells were treated with lipopolysaccharide derived from *P. gingivalis* (LPS-PG; InvivoGen, San Diego, CA, USA) at a concentration of 500 ng/mL, along with various concentrations of emodin. Wells without LPS-PG or emodin served as negative controls, while wells with only LPS-PG treatment served as positive controls. After 24 h of treatment with LPS-PG and emodin, total RNA was extracted using a Ribospin 2 kit (GeneAll Biotechnology, Hanam, Republic of Korea). Subsequently, 1 μg of RNA was used to synthesize cDNA according to the manufacturer’s instructions for the AccuPower^®^ CycleScript™ RT Master Mix (Bioneer, Daejeon, Republic of Korea). Quantitative polymerase chain reaction (qPCR) was performed using the AccuPower^®^ 2X GreenStar™ qPCR Master Mix (Bioneer). The primer sequences used for qPCR are listed in Table 1.

### 2.3. Antibacterial Activity of Emodin

The antibacterial activity of emodin was assessed using *P. gingivalis* (ATCC 53978), a key periodontal pathogen. The bacteria were cultured anaerobically in 3% brain heart infusion (BHI) broth supplemented with 50 μg/mL of hemin and 0.5 μg/mL of menadione using an anaerobic incubator (DG250 Workstation, Don Whitley Scientific, West Yorkshire, UK) under 10% H_2_, 10% CO_2_, and 80% N_2_. Following 72 h of culture, 2 mL of the bacterial suspension was transferred to 20 mL of fresh medium and cultured for an additional 72 h.

The minimum inhibitory concentration (MIC) and minimum bactericidal concentration (MBC) of emodin were determined. To determine MIC, we serially diluted emodin in a 96-well plate containing BHI broth, with a starting concentration of 200 μM. The plates were inoculated with *P. gingivalis* at an optical density of 0.1 at 600 nm and anaerobically incubated for 48 h. MIC was defined as the lowest concentration of emodin that inhibited visible bacterial growth. To determine MBC, we inoculated the bacterial suspensions used for MIC determination, and higher concentrations were inoculated onto blood agar plates and anaerobically incubated for 7 days. MBC was defined as the lowest concentration of emodin at which no bacterial colonies were observed.

### 2.4. Preparation of the Emodin-Loaded Thermoresponsive Hydrogels

**Figure 1:**
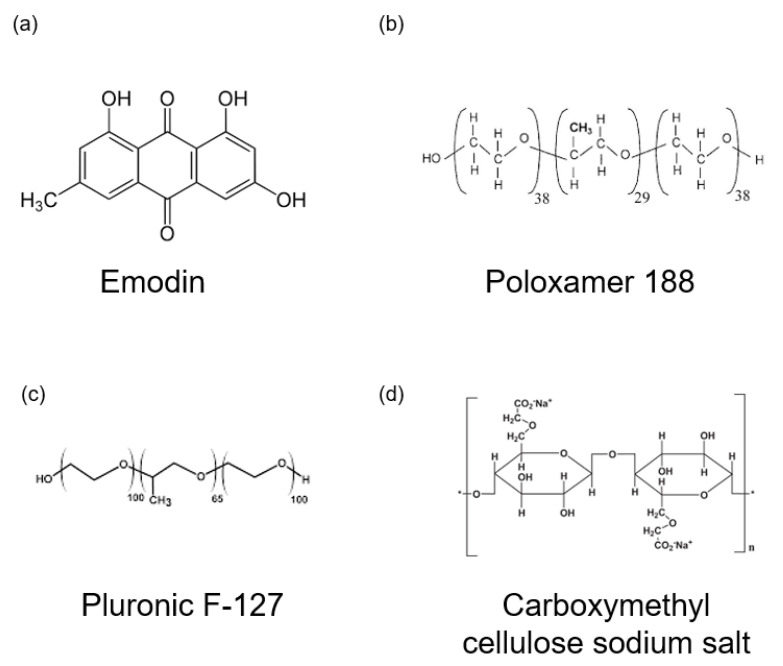
Chemical structures of gel formulation. (**a**) Emodin. (**b**) Poloxamer 188. (**c**) Pluronic F-127. (**d**) Carboxymethyl cellulose sodium salt.

For preparation of the thermoresponsive hydrogels, emodin powder was dissolved in distilled water. To modify the properties of the hydrogel, we added 1 g of poloxamer 188 (Sigma-Aldrich, St. Louis, MO, USA; average molecular weight ~8400 Da) to adjust the gelation temperature and 0.4 g of carboxymethylcellulose sodium salt (Sigma-Aldrich; low viscosity grade, Mw ~90,000 Da) to control viscosity. The mixture was stirred at room temperature for 4 h using a magnetic stirrer. Subsequently, 8 g of pluronic^®^ F-127 (Sigma-Aldrich; average Mw ~12,600 Da) and varying amounts of emodin were added to final emodin concentration of 8 mg/mL for E1 and 15.9 mg/mL for E2. The solution was stirred for 24 h at 4 °C to achieve homogeneity. The hydrogel compositions are listed in Table 2 and the chemical structures of the gel formulation are presented in Figure 1.

### 2.5. Viscosity and pH of the Emodin-Loaded Thermoresponsive Hydrogels

Viscosity of the thermoresponsive hydrogels was measured using a viscometer (DV2 RV, Brookfield, Stoughton, MA, USA) equipped with a No. 21 spindle at 1 rpm, with a starting temperature of 4 °C with subsequent increments of 5 °C up to 30 °C, followed by 1 °C increments thereafter. pH was measured using a pH meter (Orion Star A214; Thermo Fisher Scientific, Waltham, MA, USA), with calibration performed using standard pH solutions (pH 4.01, 7.00, and 11.01).

### 2.6. Scanning Electron Microscopy Findings for the Emodin-Loaded Thermoresponsive Hydrogel

The hydrogels were dropped on a sample holder and frozen at −80 °C for 15 min. After the first freeze, the hydrogel samples were cut at a 45° angle using a #11 surgical blade (Ailee, Busan, Republic of Korea) and frozen at −80 °C for 7 days. The samples were then coated with platinum under vacuum (108 auto; Cressington Scientific Instruments Ltd., Watford, UK), and the surfaces were investigated using a scanning electron microscope (JSM-6360, Jeol Ltd., Tokyo, Japan).

### 2.7. Gelation Temperature and Injectability of the Emodin-Loaded Thermoresponsive Hydrogels

To determine the gelation temperature, 5 mL of the gels was placed in glass vials and incubated in a water bath, with temperature increases of 1 °C every 5 min, starting from 20 °C. The gelation temperature was defined as the point at which no flow was observed when the vial was tilted. The injectability of the hydrogels was evaluated by loading 10 mL of the gels into syringes fitted with 23-gauge needles (SIRBAN, Hanam, Republic of Korea) and manually extruding the gel.

### 2.8. Release of Emodin from the Emodin-Loaded Thermoresponsive Hydrogels

To evaluate emodin release, 200 μL of the hydrogels was placed in 15 mm diameter glass vials and incubated at 37 °C for gelation [29]. One milliliter of phosphate-buffered saline (PBS; Cytiva, Marlborough, MA, USA) was added to the gel, and the system was incubated at 37 °C in a shaking incubator (JSSI-100C; JSR, Gongju, Republic of Korea) at 50 rpm. At specific time points, 0.9 mL of supernatant was collected and replaced with fresh PBS. The emodin concentration was quantified using a calibration curve prepared by measuring the absorbance at 260 nm using a spectrophotometer (DS-C, Denovix, Wilmington, DE, USA).

### 2.9. Cell Viability of the Emodin-Loaded Thermoresponsive Hydrogels

The cytotoxicity of the emodin-loaded hydrogels was evaluated using hPDLSCs seeded in a 96-well plate at a density of 10,000 cells/well. Hydrogel extracts were prepared using cell inserts with a 0.4 μm pore size (SPL Life Sciences, Pocheon, Republic of Korea). Specifically, 500 μL of gel was added to the cell inserts and allowed to gelate at 37 °C for 30 min. Subsequently, 500 μL of α-MEM was added to the lower wells, and the gel was incubated for 24 h to enable extraction. After 24 h, 100 μL of hydrogel extract was added to hPDLSCs for 24 h, and cell viability was measured using the CCK-8 assay.

### 2.10. Antibacterial Activity of the Emodin-Loaded Thermoresponsive Hydrogels

The antibacterial activity of emodin-loaded hydrogels was assessed using an agar diffusion assay. *P. gingivalis* ATCC 53978 was diluted to an optical density of 0.01 at 600 nm and inoculated onto blood agar plates. Paper discs (6 mm diameter) were placed on the agar, and 15 μL of hydrogel or vehicle gel was applied to the discs. Five microliters of positive (0.12% chlorhexidine) and negative (PBS) controls were also used. The plates were anaerobically incubated for 48 h, and the diameters of the inhibition zones were measured in perpendicular directions. The average diameter was then calculated.

### 2.11. Alveolar Bone Loss and Inflammatory Cytokine Expression in a Rat Model of Ligature-Induced Periodontitis

A total of 24 male Sprague–Dawley rats (Samtako, Osan, Republic of Korea) aged 7 weeks and weighing 180–220 g were used for the study. The animals were divided into four groups randomly, with six rats per group: NC, without ligature or treatment; PC, ligature without treatment; E1, ligature with E1 gel treatment; E2, ligature with E2 gel treatment. S.H.-M. was aware of the group allocation. The rats were housed in a controlled environment with a room temperature of (23 ± 2) °C and a 12 h light–dark cycle with access to food and water ad libitum. The body weights of the animals were measured every 2 days, and their bedding was replaced every 3–4 days. All animal experiments were approved by the Institutional Animal Care and Use Committee of Wonkwang University (approval no. WKU 21-95) and performed in compliance with the ARRIVE guidelines for animal research. The sample size was calculated using G*Power software version 3.1.9.7 with an effect size of 0.80.

After a 7-day acclimatization period, rats in all groups except the NC group were intraperitoneally anesthetized with chloral hydrate (400 mg/kg) to facilitate tooth ligation. For induction of periodontitis, the bilateral maxillary second molars were ligated using 4-0 silk sutures (Ailee), with the knots positioned buccally using a surgeon’s knot. Starting the day after ligation, the groups with induced periodontitis were administered 25 µL of the prepared emodin-loaded hydrogels to the ligation sites every other day under isoflurane anesthesia. Fourteen days after ligation, the animals were sacrificed using carbon dioxide.

Following sacrifice, the isolated maxillae were fixed in 4% paraformaldehyde (Geneall Biotechnology), which was replaced with PBS before micro-computed tomography (Skyscan 1076, Bruker, Billerica, MA, USA). Imaging was performed at a voltage of 100 kV, current of 100 µA, and resolution of 18 µm. The acquired sections were subjected to three-dimensional reconstruction using the CTvox program (Bruker). Bone loss was measured using ImageJ 1.8 software by calculating the distance between the cementoenamel junction (CEJ) and alveolar bone crest (ABC) on the mesial and distal aspects of the second molar. The gingival tissue surrounding the maxillary molars was carefully dissected using a #11c blade (Ailee). The samples were then homogenized in TRIzol reagent (Invitrogen, Waltham, MA, USA) to prepare for RNA extraction. Subsequently, reverse transcription qPCR (RT-qPCR) was performed to analyze gene expression levels of pro-inflammatory cytokines. The primer sequences used for qPCR are listed in Table 3. Both alveolar bone loss and RT-qPCR measurements were done triplicate and there were no exclusions.

### 2.12. Statistical Analysis

Statistical analyses were performed using IBM SPSS Statistics for Windows, Version 27.0 (IBM Corp., Armonk, NY, USA). One-way analysis of variance (ANOVA) was used to analyze cell viability (emodin and hydrogel), hydrogel pore size, pH, and breakdown of the gels, followed by Duncan’s multiple range test for post hoc comparisons. MIC of emodin, agar diffusion test results, and release of emodin from the gels were analyzed using the Kruskal–Wallis test, followed by the Mann–Whitney U test with Bonferroni correction. A two-tailed t-test was used to compare alveolar bone loss and tissue mRNA levels between groups. In all analyses, *p* < 0.05 was considered statistically significant.

## 3. Results

### 3.1. Cell Viability and Anti-Inflammatory and Antibacterial Activities of Emodin

**Figure 2:**
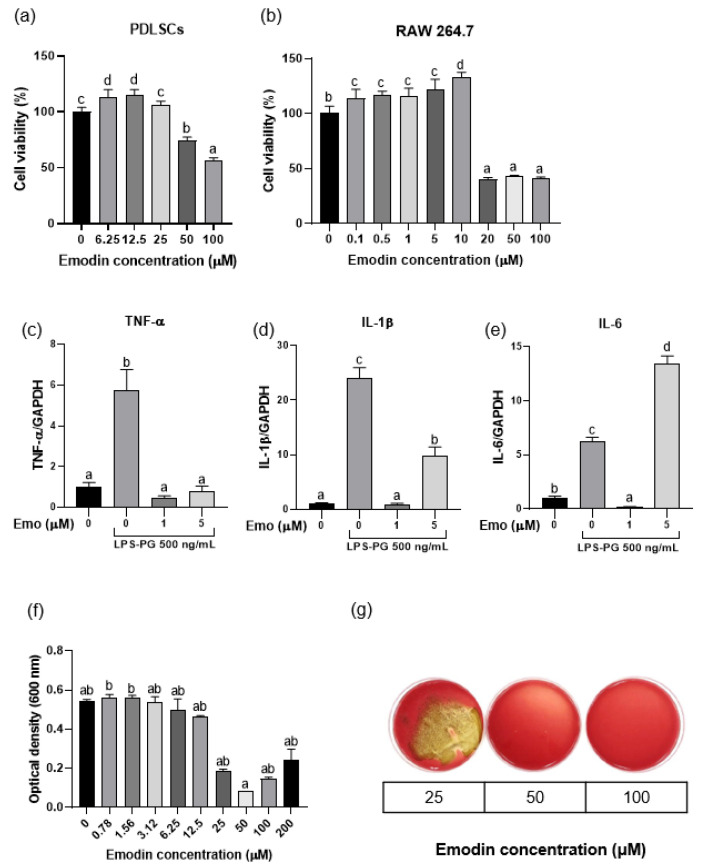
Biological effects of emodin. (**a**,**b**) Effect of emodin on the viability of periodontal ligament stem cells (PDLSCs) and RAW 264.7 cells. (**c**–**e**) Anti-inflammatory effects of emodin against lipopolysaccharide derived from *Porphyromonas gingivalis* (LPS-PG) on RAW 264.7 cells; 500 ng/mL of LPS-PG significantly increases mRNA expression of proinflammatory cytokines, while 1 μM of emodin significantly decreases mRNA expression of proinflammatory cytokines (*p* < 0.05). (**f**,**g**) The minimal inhibitory concentration and minimal bactericidal concentration of emodin against *P. gingivalis* are both 50 μM. Different lowercase letters indicate significant differences among groups.

Emodin did not exhibit cytotoxicity up to a concentration of 50 μM for hPDLSCs and 10 μM for RAW 264.7 cells, showing relative cell viability of >70% at these concentrations when compared with the negative control (Figure 2a,b). In addition, in LPS-stimulated RAW 264.7 cells, 1 μM emodin significantly reduced the mRNA levels of TNF-α, IL-1β, and IL-6 compared to the LPS-only control (*p* < 0.05; Figure 2c–e), while 5 μM emodin significantly reduced TNF-α and IL-1β. Furthermore, emodin inhibited the growth of *P. gingivalis* at concentrations ≥50 μM, which was identified as the MIC (Figure 2f). The MBC was determined to be 50 μM (Figure 2g). Collectively, these findings indicate that emodin can be used at non-cytotoxic concentrations to achieve both anti-inflammatory and antibacterial effects, supporting its potential as a therapeutic agent for periodontitis.

### 3.2. Viscosity and pH of the Emodin-Loaded Thermoresponsive Hydrogels

**Figure 3:**
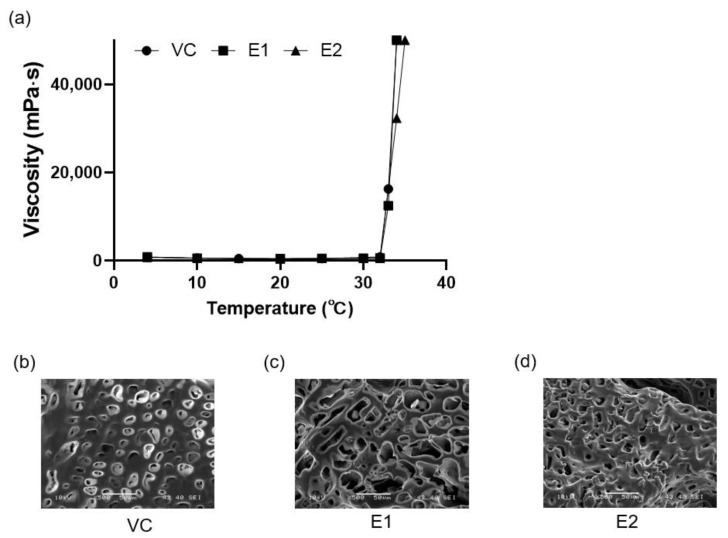
Characteristics of the thermoresponsive hydrogels with or without emodin. (**a**) Viscosities of the thermoresponsive hydrogels according to temperature. (**b**–**d**) Scanning electron microscopy (SEM) images of the thermoresponsive hydrogels. VC: vehicle control without emodin; E1 and E2: emodin-loaded hydrogels with emodin concentrations of 8 and 15.9 mg/mL, respectively.

Both the vehicle and emodin-loaded thermoresponsive hydrogels changed to a gel form at 33–34 °C and showed a sharp increase in viscosity from 32 °C (Figure 3a). The pH of the hydrogels ranged from 6.6 to 7.0, as described in Table 4. At 33–34 °C, the hydrogels underwent a sol–gel transition, meaning they would form a gel in the periodontal pocket while remaining injectable at room temperature. Additionally, the hydrogels’ pH was near neutral, indicating compatibility with physiological conditions (Table 4).

### 3.3. Scanning Electron Microscopy Analysis of the Emodin-Loaded Thermoresponsive Hydrogels

The thermoresponsive hydrogels had irregular pores (Figure 3b–d). The E1 gel showed a significantly larger pore size (16.25 ± 5.51 μm) than the VC (7.54 ± 2.91 μm) and E2 (10.50 ± 4.51 μm) gels did (*p* < 0.05).

### 3.4. Injectability and Gelation Temperature of the Emodin-Loaded Thermoresponsive Hydrogels

**Figure 4:**
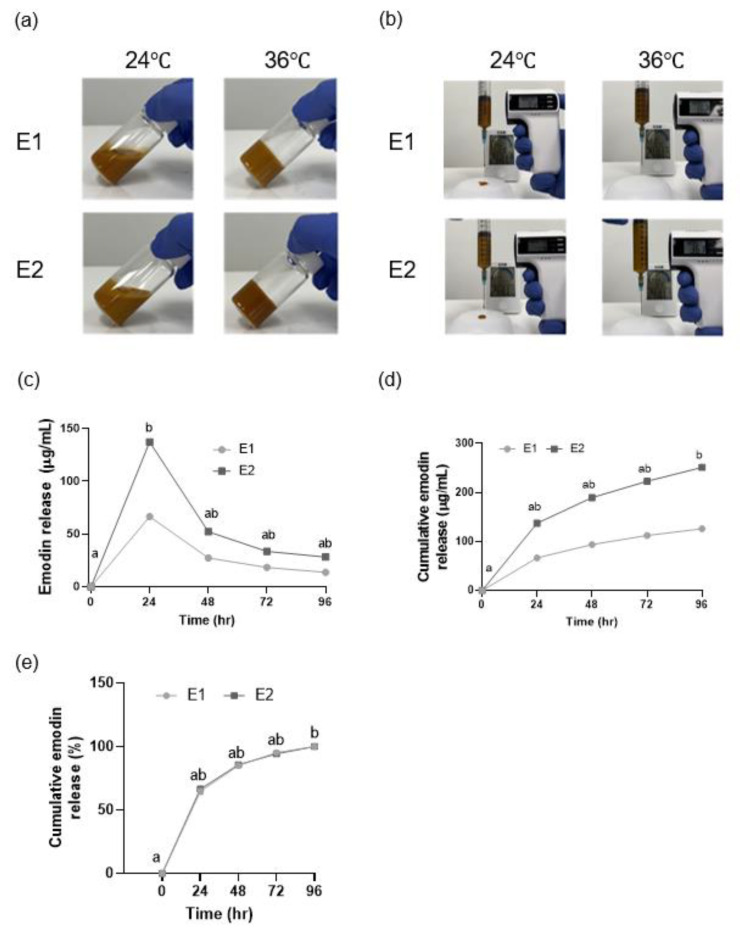
Gelation temperature, injectability, and cumulative release and breakdown of the emodin-loaded thermoresponsive hydrogels. (**a**) These images serve as a visual illustration demonstrating the sol state at room temperature and the gel state at 36 °C, as determined by the vial inversion method. (**b**) Injectability of the hydrogels. (**c**) Release of emodin from the hydrogels, shown as emodin concentrations. (**d**) Cumulative release of emodin from the hydrogels, shown as emodin concentrations. (**e**) Cumulative release of emodin from the hydrogels. The gels show an initial burst release in the first 24 h, with sustained release thereafter. E1 and E2: emodin-loaded hydrogels with emodin concentrations of 8 and 15.9 mg/mL, respectively. Different lowercase letters indicate significant differences among timepoints.

Both the E1 and E2 hydrogels were injectable through a 23-gauge needle at 24 °C and became noninjectable at 36 °C because of gelation (Figure 4a,b). The gelation temperature of both gels was <37 °C (Table 4).

### 3.5. Emodin Release from the Emodin-Loaded Thermoresponsive Hydrogels

Both the E1 and E2 gels exhibited an initial burst release of 64% within the first 24 h. The emodin release rates for E1 and E2 at 96 h were significantly different from those at 0 h (*p* < 0.05; Figure 4c–e).

### 3.6. Effect of the Emodin-Loaded Thermoresponsive Hydrogels on Cell Viability

**Figure 5:**
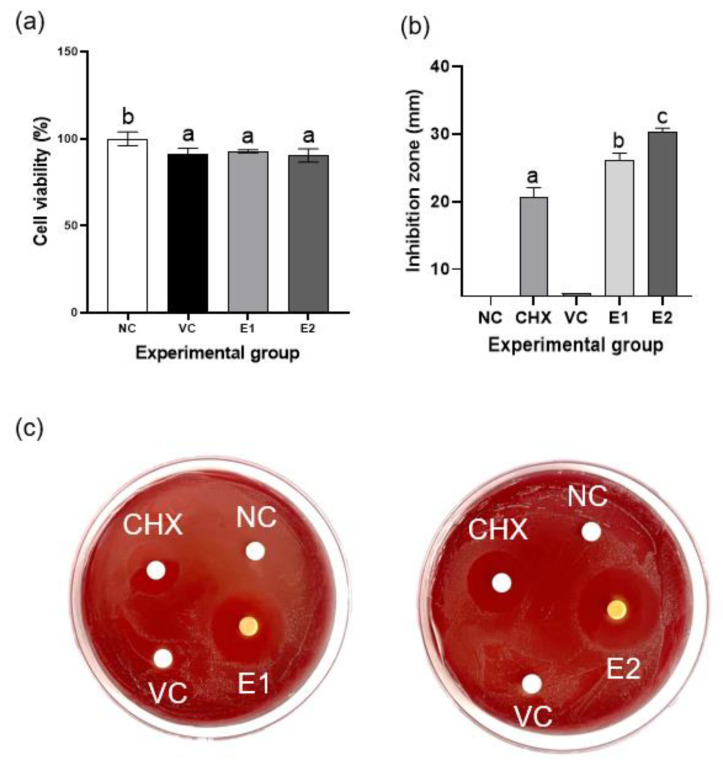
Cytotoxicity and antibacterial effect of the emodin-loaded thermoresponsive hydrogels. (**a**) Effects of the gel extracts on the viability of periodontal ligament stem cells (PDLSCs). None of them show cytotoxicity against PDLSCs. (**b**) Inhibition zones for *Porphyromonas gingivalis* in the agar diffusion test. The E1 and E2 hydrogels show significant antibacterial activity compared with that of CHX. Different lowercase letters indicate significant differences among groups. (**c**) Photographs of blood agar plates. CHX: 0.12% chlorhexidine; NC: negative control; VC: vehicle control without emodin; E1 and E2: emodin-loaded hydrogels with emodin concentrations of 8 and 15.9 mg/mL, respectively.

The viability of the hPDLSCs was 90% with the extracts from the VC, E1, and E2 groups (Figure 5a); this confirmed their nontoxic nature.

### 3.7. Antibacterial Activity of the Emodin-Loaded Thermoresponsive Hydrogels

The negative control and VC exhibited no antibacterial activity, whereas the E1 and E2 hydrogels displayed significantly higher antibacterial activity than did CHX, 0.12% chlorhexidine (*p* < 0.05); this indicated effective inhibition of the growth of *P. gingivalis* (Figure 5b–c).

### 3.8. Effects on Alveolar Bone Loss and Inflammatory Cytokine Expression in a Rat Model of Ligature-Induced Periodontitis

**Figure 6:**
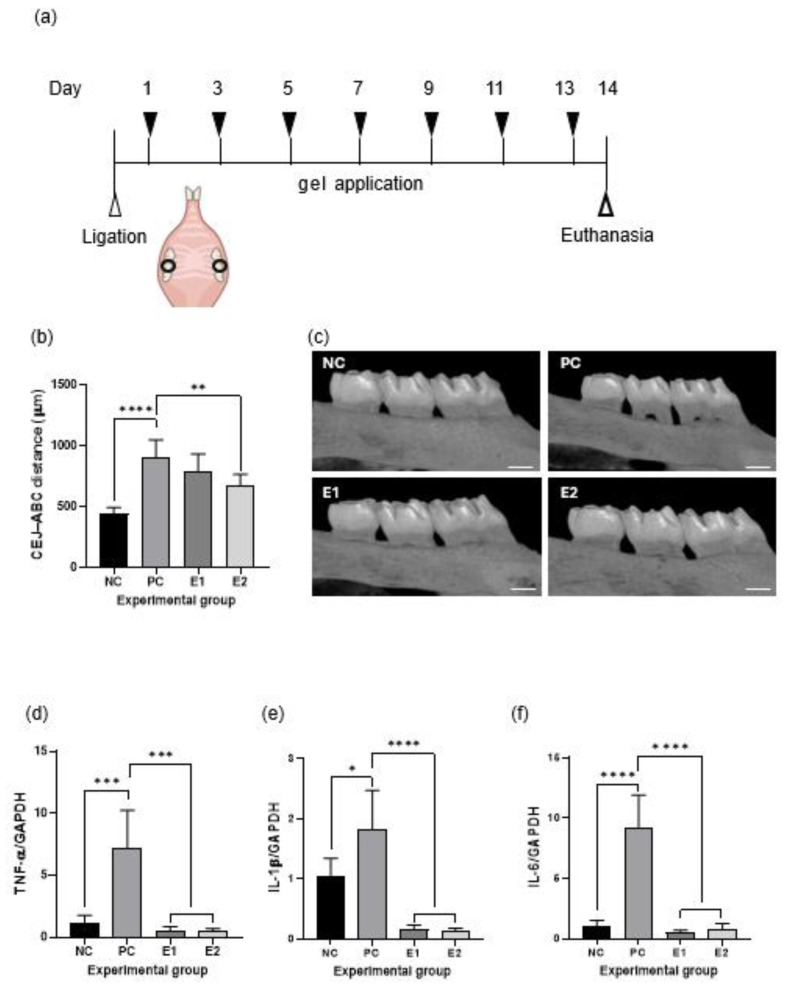
Effects of the emodin-loaded thermoresponsive hydrogels in a rat model of periodontitis. (**a**) Graphical representation of the rat model of ligature-induced periodontitis. (**b**) Alveolar bone loss in the experimental groups. (**c**) Micro-computed tomography images of the experimental groups (scale bar = 500 μm). (**d**–**f**) Anti-inflammatory effects of the emodin-loaded thermoresponsive hydrogels. Both the E1 and E2 gels significantly decrease mRNA expression of proinflammatory cytokines in gingival tissue (*p* < 0.05). Statistical analysis was performed using a two-tailed t-test (*: *p* < 0.05, **: *p* < 0.01, ***: *p* < 0.001, ****: *p* < 0.0001). NC: negative control (without ligature or treatment); PC: positive control (ligature without treatment); E1: ligature with E1 gel treatment; E2: ligature with E2 gel treatment; E1 and E2: emodin-loaded hydrogels with emodin concentrations of 8 and 15.9 mg/mL, respectively; TNF-α: tumor necrosis factor alpha; IL: interleukin; GAPDH: glyceraldehyde-3-phosphate dehydrogenase; CEJ: cementoenamel junction; ABC: alveolar bone crest.

The rat model is shown in Figure 6a. To determine the effect of the emodin-loaded thermoresponsive hydrogels on alveolar bone loss, the distance between the CEJ and ABC was measured using micro-CT (Figure 6b,c). The PC group showed significantly greater alveolar bone loss than the NC group did (*p* < 0.05). The E2 group showed significantly lesser alveolar bone loss than the PC group did (*p* < 0.05), whereas the E1 and PC groups showed no significant difference (*p* > 0.05). When RT-qPCR was performed for the maxillary gingival tissue, the expression of proinflammatory cytokines (TNF-α, IL-1β, and IL-6) in the E1 and E2 groups was significantly reduced relative to that in the PC group (*p* < 0.05; Figure 6d–f).

## 4. Discussion

This study investigated the antibacterial and anti-inflammatory effects of emodin and emodin-loaded thermoresponsive hydrogels as local drug delivery systems for the treatment of periodontitis. The physical and biological properties of the emodin-loaded thermoresponsive hydrogels were studied both in vitro and in vivo using a rat model of ligature-induced periodontitis.

Emodin reduced the levels of proinflammatory cytokines such as TNF-α, IL-1β, and IL-6 at a low concentration of 1 μM, while it showed cytotoxic effects on RAW 264.7 cells at concentrations up to 10 μM. Because RAW 264.7 cells are highly sensitive to stimuli such as LPS, inflammatory responses can be strongly induced [30]. Interestingly, increasing the emodin concentration to 5 μM did not further reduce IL-1β and IL-6 production compared to 1 μM. This suggests that 1 μM was sufficient to elicit a near-maximal anti-inflammatory response, and that higher concentrations might not confer additional benefit potentially due to mild cytotoxic stress at 5 μM [31,32,33]. Higher emodin concentrations might induce mild oxidative stress that activates pro-inflammatory pathways, therefore reducing the additional anti-inflammatory effect [32]. Low doses of anthraquinones trigger adaptive stress responses, whereas higher doses produce excess reactive oxygen species and cellular stress [31]. Emodin also demonstrated no cytotoxicity toward hPDLSCs at concentrations up to 50 μM. Previous studies have also reported that emodin exerts anti-inflammatory effects on RAW 264.7 cells through a peroxisome proliferator-activated receptor-γ-dependent pathway, further supporting its therapeutic potential [33].

Emodin showed antimicrobial activity against *P. gingivalis*, a major periodontal pathogen, with an MIC and MBC of 50 μM. Wang et al. reported lower MIC values (20–30 μM) against gram-negative periodontal pathogens, including *Fusobacterium nucleatum* and *Prevotella intermedia* [34]. In addition, emodin did not cause cytotoxicity to PDLSCs up to a concentration of 50 μM, the same concentration at which it showed bactericidal activity against *P. gingivalis*. These findings highlight the potential applicability of this hydrogel in the management of periodontitis.

Moreover, the hydrogel exhibited a rapid increase in viscosity at temperatures below that in the periodontal pocket, which is 35 °C [35]. This suggests that it can be adequately retained in the oral environment. At physiological oral temperatures, the gel underwent gelation while retaining injectability at room temperature; this makes it a promising local drug delivery system [36]. Commercially available hydrogels often have a very high viscosity, which makes injection challenging. In contrast, the emodin-loaded hydrogel developed in this study was easily injectable and effectively reduced alveolar bone loss and inflammatory marker levels in vivo [17]. However, this study did not include measurements of the hydrogel’s viscoelastic properties such as storage and loss moduli. Future work will be needed to characterize these rheological aspects in detail.

The gelation of the poloxamer-based hydrogel is assumed to be driven by physical cross-linking of polymer chains rather than chemical bonds. At low temperatures the poloxamers (Pluronic F-127 and Poloxamer 188), which are amphiphilic PEO-PPO-PEO triblock copolymers, remain freely soluble. However at approximately 35–37 °C, their hydrophobic PPO blocks aggregate into micelles, which then pack into a physically entangled gel network [37,38]. CMC, a hydrophilic polymer that is not thermoresponsive, interacts with poloxamers through hydrogen bonding and chain entanglement, thereby enhancing viscosity and contributing to the stabilization of the gel structure [39]. These dual mechanisms, micelle aggregation and polymer–polymer hydrogen bonding, contribute to the gel’s stability and injectability under physiological conditions [40].

The release profiles of the E1 and E2 hydrogels demonstrated an initial burst release of emodin within the first 24 h, followed by sustained release over 96 h, ultimately reaching 100% cumulative release. This controlled release pattern suggests that the hydrogel maintains a stable concentration of emodin at the target site within the periodontal pocket. Although scanning electron microscopy revealed differences in pore size (7–16 μm) among the hydrogel formulations, these morphological variations did not result in any noticeable differences in the drug release profiles. This indicates that the release of emodin was primarily affected by the polymer matrix erosion and micellar diffusion rather than by the static pore structure.

In the animal study, compared with the NC group, the PC group showed alveolar bone loss, which indicated effective induction of periodontitis. Notably, the E2 group demonstrated a significant reduction in alveolar bone loss relative to that in the PC group; this indicated the in vivo therapeutic potential of the hydrogel against periodontitis. Luo et al. found that emodin prevented inflammatory bone loss by significantly inhibiting osteoclast activity, thus preserving bone mass and structure [41]. Considering the difference between the E1 and E2 gels, the E2 gel demonstrated significantly greater efficacy in reducing alveolar bone loss than did the E1 gel, even though both formulations showed comparable anti-inflammatory effects. This concentration-dependent efficacy may be explained by the pharmacokinetics of local drug delivery in the periodontal pocket. The higher emodin concentration in the E2 gel likely established a steeper diffusion gradient, allowing therapeutic levels to penetrate deeper into the periodontal tissues and maintain effective concentrations for a longer duration, despite similar release profiles in vitro. In addition, the antibacterial activity of emodin appeared to exhibit a dose-dependent relationship with bacterial growth inhibition, as evidenced by the agar diffusion test. From a clinical perspective, these findings suggest that higher emodin concentrations may be necessary to achieve optimal therapeutic effects, particularly for the prevention of alveolar bone resorption.

In the rat model of ligature-induced periodontitis, expression levels of the proinflammatory cytokines TNF-α, IL-1β, and IL-6 in gingival tissue were significantly reduced in the E1 and E2 groups compared with those in the PC group. Previous studies have identified TNF-α, IL-1β, and IL-6 as key proinflammatory cytokines involved in periodontal tissue destruction and systemic disease progression [42,43]. Collectively, these findings strongly support the dual capacity of emodin to attenuate inflammation and prevent alveolar bone loss. It should also be noted that a vehicle hydrogel without emodin was not included in the in vivo study, based on the in vitro results showing that the vehicle hydrogel had no effect by itself. Therefore, the observed therapeutic effects are primarily attributed to the release of emodin from the formulation.

This study focused on two gel formulations containing different emodin concentrations. However, future research should explore the optimal pH required to enhance the compatibility of the hydrogel with the oral environment. In addition, a systematic investigation of the hydrogel’s dissolution and degradation behavior will be important for understanding its long-term performance under physiological conditions. Further histological studies are required to assess potential systemic toxicity resulting from the initial burst release of emodin in vivo.

In summary, emodin exhibited antibacterial effects against *P. gingivalis* (ATCC 53978) without cytotoxic effects on normal cells in vitro. In vivo, the application of emodin-loaded thermoresponsive hydrogels effectively reduced alveolar bone loss and proinflammatory cytokine levels. These findings suggest that the emodin-loaded thermoresponsive hydrogel is a potential novel therapeutic agent for the treatment of periodontitis, providing a strong foundation for further development and clinical translation with the goal of improving periodontal health. Future studies should focus on optimizing the gel formulation, performing histological analysis, and exploring combination therapies with conventional mechanical debridement to enhance clinical translation.

## 5. Conclusions

This study highlights the potential of emodin-loaded thermoresponsive hydrogels as innovative therapeutic agents for periodontitis. These findings provide a strong foundation for further development and clinical translation, with the goal of improving periodontal health.

## Figures and Tables

**Table 1:** Primer sequences used in the study for in vitro RT-qPCR.

Gene	Forward Primer	Reverse Primer
GAPDH	GCCTCCTCCAATTCAACCCT	ATCCGTTCACACCGACCTTC
IL-1β	GCCACCTTTTGACAGTGATGAG	AAGGTCCACGGGAAAGACAC
IL-6	CAACGATGATGCACTTGCAGA	TGTGACTCCAGCTTATCTCTTGG
TNF-α	ACCCTCACACTCACAAACCA	ATAGCAAATCGGCTGACGGT

**Table 2:** Formulation of the thermoresponsive hydrogels used in this study.

Code	P188 (g)	CMC (g)	PF127 (g)	Emodin (g)	Distilled Water (mL)	Emodin Concentration (mg/mL)
VC	1	0.4	8	0	40	0
E1	1	0.4	8	0.4	40	8.0
E2	1	0.4	8	0.8	40	15.9

**Table 3:** Primer sequences used in the study for in vivo RT-qPCR.

Gene	Forward Primer	Reverse Primer
GAPDH	GGCCTTCCGTGTTCCTA	AAGGTGGAAGAATGGGAGTTG
IL-1β	TGTGATGAAAGACGGCACAC	CTTCTTCTTTGGGTATTGTTTGG
IL-6	ACAAGTCCGGAGAGGAGACT	ACAGTGCATCATCGCTGTTC
TNF-α	ATGGCCCAGACCCTCACACTCAGA	CTCCGCTTGGTGGTTTGCTACGAC

**Table 4:** pH values and gelation temperatures for the hydrogels used in this study.

Code	pH	Gelation Temperature (°C)
VC	6.92 ± 0.01 ^b^	34
E1	6.63 ± 0.01 ^a^	34
E2	6.61 ± 0.01 ^a^	33

Different lowercase letters indicate significant differences among groups.

## Data Availability

The original contributions presented in the study are included in the article, further inquiries can be directed to the corresponding author.
